# Identification of diabetic neuropathic patients at risk of foot ulceration through finite element models and cluster analysis

**DOI:** 10.1186/1757-1146-7-S1-A27

**Published:** 2014-04-08

**Authors:** Annamaria Guiotto, Zimi Sawacha, Alessandra Scarton, Gabriella Guarneri, Angelo Avogaro, Claudio Cobelli

**Affiliations:** 1 Department of Information Engineering, University of Padova, Padova, 35131, Italy; 2 Department of Clinical Medicine and Metabolic Disease, University Polyclinic, Padova, 35128, Italy

## Background

Diabetic foot is an invalidating complication of diabetes mellitus that can lead to foot ulceration and amputations. While experimental analyses are limited solely to measurements of interfacial variables, three-dimensional (3D) patient specific finite element models (FEMs) of the foot can provide both the interfacial pressures and insight into internal stresses and strains tolerated by the plantar tissue [1]. FEMs allows quantifying the loads developed in the different anatomical structures of the foot and to understand how these affect foot tissue [2]. The aim of this study was to identify the neuropathic subjects at risk of ulceration with a cluster analysis classification of simulated plantar pressures and internal stresses. Simulations were ran with gait analysis data acquired 5 years prior to ulcerations.

## Methods

Foot biomechanical analysis was carried out as in [3] on 16 diabetic neuropathic subjects by measn of a 6 cameras motion capture system (BTS, Padova), integrated and synchronized with 2 force plates (Bertec, USA), 2 plantar pressures systems (Imagortesi, Piacenza). For each patient the 3D kinematics, ground reaction forces and plantar pressures were calculated. Six of these subjects developed ulcers under metatarsals heads within 5 years after the acquisitions (ulcerated subjects (US)- age 62.3±4.1 years, BMI 26.3±2.0 kg/m^2^) while the other ten did not (non US - age 63.2±6.4 years, BMI 24.3±2.9 kg/m^2^).

**Figure 1:**
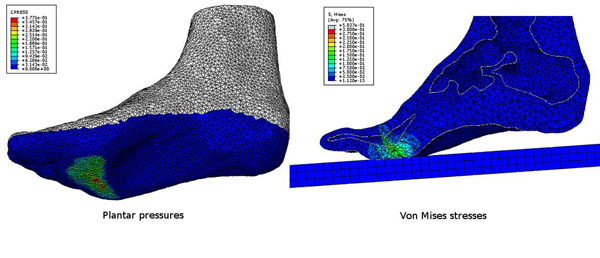
Example of the results of a FEM simulation with ulcerated subject’s boundary conditions: Plantar pressures (left) and Von Mises internal stresses (right).

In order to obtain the internal stresses (Von Mises and principal stresses) and the simulated plantar pressures (Figure 1), a recently developed 3D FEM [4] was adopted and the simulations were run adopting the experimental kinematic and kinetics as boundary conditions as in [4]. The midstance and the push-off phases of gait were considered as they are the instants when critical loads occur in the forefoot of the diabetic subjects.

K-means and hierarchical cluster analysis were performed as in [5] with simulated plantar pressures and/or internal stresses as input.

## Results

The hierarchical method (Ward’s linkage) which led to the definition of three clusters (Table 1) gave the best result: 5 US were included in one cluster with only 3 non US.

**Table 1:** Results of the hierarchical cluster analysis: 3 clusters. Values are normalized over the subject’s weight. PP=plantar pressure.

Cluster	N° subjects ulcer/no ulcer	Push-off			Mid-stance		
		**Peak PP**	**Mean PP**	**Von Mises**	**Peak PP**	**Mean PP**	**Von Mises**

1	0 / 3	0.423	0.108	0.000381	0.323	0.080	0.000251
		
		0.052	0.023	0.000174	0.020	0.010	0.000044

2	1 / 4	0.371	0.097	0.000348	0.275	0.060	0.000245
		
		0.041	0.019	0.000050	0.017	0.007	0.000024

3	5 / 3	0.390	0.104	0.000378	0.277	0.062	0.000228
		
		0.035	0.007	0.000042	0.028	0.004	0.000021

## Conclusions

A longer follow up is needed in order to verify whether the neuropathic subjects in cluster 2 and 3 will develop ulcers. A larger dataset is needed to further validate this methodology. Besides these limitations, results showed that combined FEMS and cluster analysis allowed to infer useful informations on the risk of ulceration even five years prior to the wound evolution.
